# A Regulatory Role of NAD Redox Status on Flavin Cofactor Homeostasis in *S. cerevisiae* Mitochondria

**DOI:** 10.1155/2013/612784

**Published:** 2013-09-01

**Authors:** Teresa Anna Giancaspero, Vittoria Locato, Maria Barile

**Affiliations:** ^1^Istituto di Biomembrane e Bioenergetica, CNR, Via Orabona 4, 70126 Bari, Italy; ^2^Centro Integrato di Ricerca, Università Campus Bio-Medico di Roma, Via Alvaro del Portillo 21, 00128 Roma, Italy; ^3^Dipartimento di Bioscienze, Biotecnologie e Biofarmaceutica, Università degli Studi di Bari “Aldo Moro”, Via Orabona 4, 70126 Bari, Italy

## Abstract

Flavin adenine dinucleotide (FAD) and nicotinamide adenine dinucleotide (NAD) are two redox cofactors of pivotal importance for mitochondrial functionality and cellular redox balance. Despite their relevance, the mechanism by which intramitochondrial NAD(H) and FAD levels are maintained remains quite unclear in *Saccharomyces cerevisiae*. We investigated here the ability of isolated mitochondria to degrade externally added FAD and NAD (in both its reduced and oxidized forms). A set of kinetic experiments demonstrated that mitochondrial FAD and NAD(H) destroying enzymes are different from each other and from the already characterized NUDIX hydrolases. We studied here, in some detail, FAD pyrophosphatase (EC 3.6.1.18), which is inhibited by NAD^+^ and NADH according to a noncompetitive inhibition, with *Ki* values that differ from each other by an order of magnitude. These findings, together with the ability of mitochondrial FAD pyrophosphatase to metabolize endogenous FAD, presumably deriving from mitochondrial holoflavoproteins destined to degradation, allow for proposing a novel possible role of mitochondrial NAD redox status in regulating FAD homeostasis and/or flavoprotein degradation in *S. cerevisiae*.

## 1. Introduction

Flavin adenine dinucleotide (FAD) and nicotinamide adenine dinucleotide (NAD) are two molecules of pivotal importance for mitochondrial functionality, given their role as redox cofactors of a large number of dehydrogenases, reductases, and oxidases mainly involved in energy production and redox homeostasis [1–4]. Additional emerging regulatory roles are linked to a number of additional cofactor-dependent events, such as protein folding, apoptosis, gene silencing, transcriptional regulation, DNA repairs and calcium-dependent signaling pathways. In many of these processes NAD and FAD are also involved in nonredox reactions (for recent reviews see [2, 5, 6]). In particular, NAD homeostasis and NAD-dependent modification of target proteins play a crucial role in calorie-restriction-induced life-span extension and in age-related metabolic diseases [7–9]. Consistently, NAD- and FAD-dependent enzymes deficiency and/or impairment in flavin and NAD homeostasis in humans and experimental animals have been linked to several diseases, such as cancer, cardiovascular diseases, anemia, abnormal fetal development, and different neuromuscular and neurological disorders [10–14].

The relevance of such processes merits further research aimed to better describe NAD and FAD homeostasis and flavoenzyme biogenesis, especially in those organisms that can be simple and suitable model for human diseases. The conserved biological processes with all eukaryotic cells, together with the possibility of simple and quick genetic manipulation, allowed for proposing the budding yeast, *Saccharomyces cerevisiae*, as the premier model to understand the biochemistry and molecular biology of mammalian cells and to decipher molecular mechanisms underlying human diseases [15–17].

In yeast and most other organisms, besides *de novo* synthesis of NAD, the regeneration of NAD from its nicotinamide degradation products has been described in some detail [8, 18, 19]. This salvage pathway accompanies NAD^+^-dependent signaling processes which, differently from those in which NAD works as redox cofactor, require constant replenishment of cellular NAD pools. NAD^+^ salvage pathway takes place in the nucleus [20]. Differently from mammals [21, 22], NAD^+^ is not synthesized in yeast mitochondria; consistently, two mitochondrial carriers (NDT1/2) seem to be responsible for replenishing mitochondrial NAD^+^ level in yeasts [23, 24].

As regards NAD^+^ into nicotinamide degradation products conversion, which occurs in many NAD^+^-dependent signaling processes, the NAD^+^ glycoside bound is potentially cleaved via reactions catalyzed by transferases (EC 2.4.2.-), like poly(ADP-ribose)polymerase; deacetylases (EC 3.5.1.-), like sirtuins; or hydrolases (EC 3.2.2.5) to produce nicotinamide and a variety of ADP-ribosyl products [5, 25]. From this wide spectrum of NAD^+^ consuming enzymes, only sirtuins (SIRTs) have been identified in yeasts [26]. No member, out of the five *S. cerevisiae* sirtuins, seems to be localized into mitochondria.

Another way to cleave the pyridine nucleotide molecule is at the level of pyrophosphate bond via hydrolytic enzymes. A diphosphatase (pyrophosphatase), performing an enzymatic activity towards NADH, as preferred substrate, and giving NMNH and AMP as products, has been characterized in yeast as belonging to the NUDIX hydrolase family (EC 3.6.1.-) [27]. This enzyme, namely, Npy1p encoded by *YGL067W*, is able to catalyze NAD^+^ hydrolysis and also very weakly FAD hydrolysis; it was reported to be located in peroxisomes, active at alkaline pH, and strongly inhibited by F^−^. The existence of a mitochondrial isoenzyme has not been reported so far [28].

Turning to FAD, it is synthesized starting from riboflavin (Rf, vitamin B_2_) via the sequential action of riboflavin kinase or ATP: riboflavin 5′-phosphotransferase (RFK, EC 2.7.1.26) and FAD synthase or FMN: ATP adenylyltransferase (FADS, EC 2.7.7.2). The first eukaryotic genes encoding for RFK [29] and FADS [30] have been identified and cloned in *S. cerevisiae*. Besides in the cytosol, FAD synthesis occurs also in yeast mitochondria [31, 32], thus necessitating Rf uptake into the organelle. The same mitochondrial pathway operates also in mammals and plants [33–35].

The knowledge of FAD cleavage events in yeast is rather poor, as opposed to its biosynthesis. The first investigation on FAD hydrolysis was carried out on cellular extracts obtained by the flavinogenic yeast *Pichia guilliermondii* [36, 37]. Following the demonstration of the existence of a mitochondrial FAD pyrophosphatase (FADppase, EC 3.6.1.18) and FMN phosphohydrolase (EC 3.1.3.2) in rat liver mitochondria [38], further functional evidence of FAD cleaving enzymes has been obtained in *S. cerevisiae* mitochondria (SCM) [39, 40]. The molecular identification of FADppase is still lacking, while a gene encoding for a specific FADppase so far has been cloned and identified in *Arabidopsis* and named *AtNUDX23*. It belongs to the NUDIX hydrolase family and is distributed in plastids [41, 42]. The possibility that some members of *S. cerevisiae* NUDIX hydrolase family were able to hydrolyze FAD remains to be investigated, in the frame of characterization of a putative mitochondrial FADppase [40].

Here we studied the ability of SCM to catalyze NAD and FAD hydrolysis via enzymatic activities which are different from the already characterized NUDIX hydrolases. The differential inhibition by the oxidized and reduced form of NAD, together with the ability of mitochondrial FADppase to metabolize endogenous FAD, presumably deriving from mitochondrial holoflavoproteins destined to degradation, allows for proposing a novel possible role of mitochondrial NAD redox status in regulating FAD homeostasis and, possibly, flavoprotein degradation in *S. cerevisiae*.

## 2. Materials and Methods

### 2.1. Materials

All reagents and enzymes were from Sigma-Aldrich (St. Luis, MO, USA), Zymolyase was from ICN (Abingdon, UK), and Bacto Yeast Extract was from Difco (Franklin Lakes, NJ, USA). Mitochondrial substrates were used as Tris salts at pH 7.0. Solvents and salts used for HPLC were from J.T.Baker (Center Valley, PA, USA).

### 2.2. Yeast Strain, Media, and Growth Conditions

The wild-type *S. cerevisiae* strain (EBY157A, genotype *MAT*α* ura 3–52 MAL2-8* ^*c*^ *SUC2 p426MET25*) used in this work was derived from the CEN.PK series of yeast strain and was obtained from P. Kotter (Instituet fuer Mikrobiologie, Goethe-Universitaet Frankfurt, Frankfurt, Germany), as already described in [31]. Cells were grown aerobically at 28°C with constant shaking in a semisynthetic liquid medium (3 g/L yeast extract, 1 g/L KH_2_PO_4_, 1 g/L NH_4_Cl, 0.5 g/L NaCl, 0.5 g/L CaCl_2_·2H_2_O, MgCl_2_·6H_2_O, 20 mg/L uracil, 0.05% glucose) supplemented with 2% ethanol as carbon source. The pH of the medium was adjusted to 5.5 with HCl.

### 2.3. Mitochondria Isolation and Purification

Crude *S. cerevisiae *mitochondria (SCM) were isolated according to [31]. For pure SCM preparation, the final mitochondrial pellet was resuspended in the isolation medium, consisting of 0.6 M Mannitol and 20 mM HEPES pH 7.4, to obtain 5 mg mitochondrial protein/mL and subsequently purified from extramitochondrial contaminations using a sucrose gradient basically as described in [43]. The intactness of mitochondrial inner membrane was checked measuring the latency of release of the mitochondrial matrix enzyme fumarase (FUM) as in [34, 44] by treating SCM with the nonionic detergent Triton X-100 (TX100, 0.1%) at 0°C for 1 min. The mitochondrial functionality was assessed by oxygen uptake measurements carried out using a Gilson oxygraph as in [31]. The SCM purity was checked by measuring in SCM and spheroplasts (sphero) the activities of different marker enzymes such as cytosolic pyruvate decarboxylase (PDC), vacuolar alkaline phosphatase (AP), peroxisomal D-aminoacid oxidase (D-AAOX), and mitochondrial citrate synthase (CS) via spectrophotometric assays, essentially as described in [31, 45]. Mitochondrial protein concentration was determined according to [46].

### 2.4. FAD Hydrolysis

FAD externally added or endogenous FAD metabolism in *S. cerevisiae* was monitored by means of fluorimetric and HPLC measurements, essentially as in [38, 47]. In the case of fluorimetric measurements, flavin derivative emission spectra (excitation wavelength at 450 nm) and time drive measurements (excitation and emission wavelengths at 450 nm and 520 nm, resp.) were carried out at 25°C in 2 mL of a standard medium consisting of 0.6 M Mannitol, 50 mM TRIS-HCl, pH 7.5, and 5 mM MgCl_2_ by means of a LS50B Perkin Elmer spectrofluorimeter. Flavin fluorescence emission spectra were corrected as in [48] by adding a few crystals of sodium dithionite to the mitochondrial suspension. When externally added FAD hydrolysis was measured, the endogenous flavin fluorescence was not considered since it was found to be negligible compared to that measured in subcellular suspension.

In each experiment, FAD, FMN, and Rf fluorescence was calibrated individually using standard solutions whose concentrations were calculated by using *ε*_450_ of 12.2 mM^−1^ · cm^−1^ for FMN and Rf, and 11.3 mM^−1^ · cm^−1^ for FAD. Under the experimental conditions used here, FAD fluorescence constant (*K*_FAD_) proved to be about ten times lower than those of FMN and Rf (*K*_FMN/Rf_) which did not differ significantly from each other [33, 49]. Thus, the rate of FAD hydrolysis, that is, the rate of FMN + Rf formation, expressed as nmol FAD hydrolyzed · min^−1^ · mg^−1^ protein, was calculated from the rate of fluorescence increase, measured as tangent to the initial part of the experimental curve by applying the following equation:

(1) $$\begin{array}{c} v_{o}=\frac{\left(\Delta F/\Delta K\times V_{f}\right)}{\Delta t\times m}, \end{array}$$

where Δ*F* is expressed in fluorescence arbitrary units, *V*_*f*_ is expressed in mL, Δ*K* = *K*_FMN/RF_ − *K*_FAD_ is expressed as *μ*M^−1^, Δ*t* is expressed in min, and *m* is the mass of protein in mg.

In the case of HPLC measurements, from aliquots of the subcellular suspension (100 *μ*L), taken at various times, perchloric extracts were obtained and neutralized with KOH [33]. FAD, FMN, and Rf were analyzed by means of HPLC, as in [31, 47]. FAD, FMN, and Rf were measured in each experiment, with a quantitative determination carried out by measuring peak areas by means of a calibration curve. It should be noted that, in externally added FAD experiments, the contribution of endogenous flavins to the measured chromatographic peak area was proved to be negligible with respect to that due to flavin derivatives found in the subcellular suspension. Since the rate of FMN/Rf appearance measured fluorimetrically *o* via HPLC showed no significant difference, these experimental approaches were used in this work indifferently.

### 2.5. NADH Oxidation and Hydrolysis

NADH metabolism in *S. cerevisiae* was monitored by means of fluorimetric measurements, using a LS50B Perkin Elmer spectrofluorimeter. NADH excitation spectra (emission wavelength at 456 nm) and time drive measurements (excitation and emission wavelength pairs at 260/456 and 340/456 nm, resp.) were carried out at 25°C in 2 mL of a standard medium.

NADH oxidation to give the nonfluorescence NAD^+^ can be revealed (when it is the sole process responsible for NADH disappearance) as a fluorescence decrease at 260/456 nm and 340/456 nm as tangent to the linear part of the experimental curve as

(2) $$\begin{array}{c} v_{\text{ox}}=\left[\frac{\left(\Delta F_{260/456}/\Delta t\right)}{K_{\text{NADH}260}}\right]\times V_{f}=\left[\frac{\left(\Delta F_{340/456}/\Delta t\right)}{K_{\text{NADH}340}}\right]\times V_{f}, \end{array}$$

where Δ*F*_ecc/em_ is expressed in fluorescence arbitrary units, *V*_*f*_ is expressed in mL, *K*_NADH260_ and *K*_NADH340_ are expressed as *μ*M^−1^, and Δ*t* is expressed in min.

Differently from oxidation, NADH hydrolysis results in products which are not fluorescent at 260/456 nm but still fluorescent at 340/456 nm (with a constant value 1.25-fold lower). Therefore, when hydrolysis is the sole process responsible for NADH disappearance, it can be revealed as a fluorescence decrease at 260/456 nm and 340/456 nm as tangent to the linear part of the experimental curve as

(3) $$\begin{array}{c} v_{\text{idr}}=\left[\frac{\left(\Delta F_{260/456}/\Delta t\right)}{K_{\text{NADH}260}}\right]\times V_{f}=\left[\frac{\left(\Delta F_{340/456}/\Delta t\right)}{\Delta K_{340}}\right]\times V_{f} \end{array}$$

with Δ*F*_260/456_/Δ*t* higher than Δ*F*_340/456_/Δ*t*.

Rearranging (2) and (3), it is possible to calculate the two components of the NADH disappearance in a complex system, where oxidation is significantly reduced with respect to hydrolysis (i.e., in the presence of KCN 1 mM).

The rate of NADH hydrolysis in the presence of residual NADH oxidation, expressed as nmol NADH hydrolyzed · min^−1^ · mg^−1^ protein, can be calculated by applying the following equation:

(4) vidr=[(ΔF260/456/Δt)/KNADH340−(ΔF340/456/Δt)/KNADH260]KNADH260·(KNADH340−ΔK340)·Vf

with Δ*F*_260/456_/Δ*t* higher or equal to Δ*F*_340/456_/Δ*t*.

The rate of NADH oxidation in the mixed system can be calculated as

(5) vox=[(ΔF340/456/Δt)/KNADH260−(ΔF260/456/Δt)/ΔK340]KNADH260·(KNADH340−ΔK340)·Vf.

In each experiment, the *K*_NADH260_ and *K*_NADH340_ values were determined by calibrating NADH fluorescence with standard solutions, whose concentrations were spectrophotometrically defined (*ε*_340_ was 6.2 mM^−1^ · cm^−1^). NMNH fluorescence was calibrated using a standard curve produced by incubating NADH (at concentrations ranging from 1 to 10 *μ*M) with excess amounts of commercial nucleotide pyrophosphatase (EC 3.6.1.9) to determine the Δ*K*_340_ value.

### 2.6. NAD^+^ Hydrolysis

NAD^+^ hydrolysis was measured by fluorimetrically monitoring the hydrolysis of nicotinamide 1,N^6^-ethenoadenine dinucleotide (*ε*-NAD^+^), essentially as described in [50, 51]. Fluorescence measurements were performed using a Perkin Elmer LS5 spectrofluorimeter (excitation and emission wavelengths set at 310 nm and 410 nm) at 25°C in 2 mL of a standard medium. The fluorescence changes produced by subcellular suspensions were calibrated by using a standard curve produced by incubating *ε*-NAD^+^ (at concentrations ranging from 1 to 6 *μ*M) with excess amounts of commercial NADase [50]. The concentration of *ε*-NAD^+^ was determined by the conversion of *ε*-NAD^+^ to *ε*-NADH using the commercial alcohol dehydrogenase reaction and assuming a molar extinction coefficient for *ε*-NADH of 6.2 mM^−1^ · cm^−1^ at 340 nm.

### 2.7. Kinetic Data Analysis

Data fitting was performed according to the Michaelis-Menten equation:

(6) $$\begin{array}{c} v=V_{\max}\cdot \frac{\left[S\right]}{K_{m}}+\left[S\right]. \end{array}$$

To fit the experimental data and to obtain estimates of the kinetic parameters use was made of the GraFit software (v. 3.00, 1992, by R. J. Leatherbarrow, Erithacus Software).

### 2.8. Statistical Analysis

All experiments were repeated at least 3 times with different mitochondrial preparations. Results are presented as mean ± standard deviation. Statistical significance was evaluated by Student's *t*-test. Values of *P* < 0.05 were considered statistically significant.

## 3. Results and Discussion

**Figure 1:**
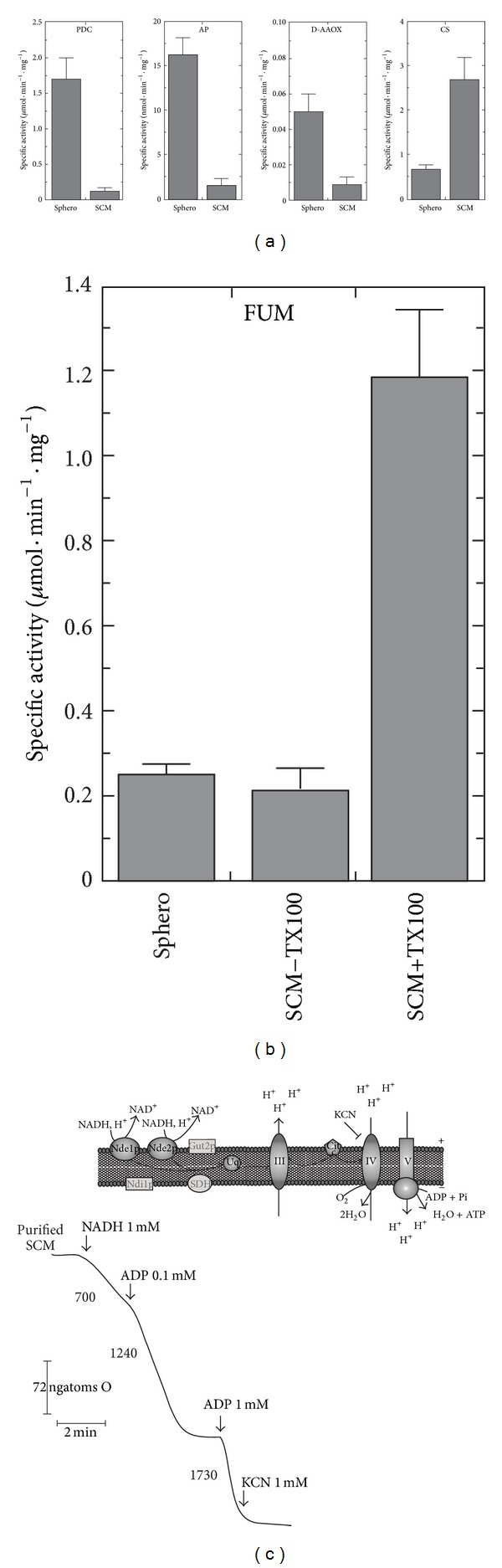
Purity, integrity, and functionality of SCM. (a) The amount of PDC, AP, D-AAOX, and CS activities in sphero and SCM (0.05–0.1 mg protein) were measured, as reported in Section 2. The values of the enzymatic activities are the mean (±SD) of three experiments performed with different cellular preparations. (b) FUM activity was measured in sphero, intact SCM (SCM − TX100), and solubilized SCM (SCM + TX100) (0.05 mg protein each) as reported in Section 2. The values are the mean (±SD) of three experiments performed with different cellular preparations. (c) A schematic representation of the electron transport along the respiratory chain starting from the external NADH dehydrogenases (*Nde1p* and *Nde2p*) is reported. Polarographic measurements of the NADH-dependent oxygen uptake rate in SCM (0.1 mg protein) were carried out as described in Section 2. The arrows indicate when the additions were made. The numbers along the trace refer to the oxygen uptake rate expressed as ngatoms O · min^−1^ · mg^−1^ mitochondrial protein.

In order to study NAD(H) mitochondrial metabolism we isolated functionally active SCM, as described before [31, 32], and further purified them, basically as in [43]. To control that SCM were significantly depleted by extramitochondrial contaminations; in Figure 1(a) we measured the distribution of the cytosolic marker enzyme (pyruvate decarboxylase, PDC), the vacuolar marker enzyme (alkaline phosphatase, AP), and the peroxisomal marker enzyme (D-aminoacid oxidase, DAAOX), with their specific activities being about tenfold (for the cytosolic and vacuolar markers) and fivefold (for the peroxisomal marker) lower in the mitochondrial fraction than those in spheroplasts (sphero). The purity of mitochondrial preparations was also assessed, by following the enrichment of the mitochondrial matrix marker enzymes citrate synthase (CS) in Figure 1(a) and fumarase (FUM) in Figure 1(b) whose specific activities were about sixfold enriched in the mitochondrial fraction. The intactness of mitochondrial suspension was also checked by following the latency of release of the mitochondrial matrix enzyme FUM, following the rupture of the inner mitochondrial membrane by Triton X-100 (TX100). An integrity of 81 ± 3% was measured in three different mitochondrial preparations (Figure 1(b)), as described in Section 2. Then, in the frame of assessing mitochondrial functionality, NADH (1 mM) was externally added to purified SCM, to induce respiration, via the two external NADH dehydrogenases (namely, Nde1p and Nde2p), as schematized in Figure 1(c). In the typical experiment reported, SCM respired NADH (1 mM) with a rate equal to 700 ngatoms O · min^−1^ · mg^−1^ protein. When ADP (0.1 mM) was added, the oxygen uptake rate increased up to 1240 ngatoms O · min^−1^ · mg^−1^ protein, with a respiratory control index (RCI) value equal to 1.8. Under this ADP limiting concentration a P/O value equal to 1.4 was measured. Addition of ADP at higher concentration (1 mM) increased the oxygen consumption rate up to 1730 ngatoms O · min^−1^ · mg^−1^ protein, with a RCI value equal to 2.5. In three experiments, performed with different mitochondrial preparations, SCM showed RCI values ranging from 2.0 to 3.0. As expected, NADH-induced respiration was almost totally inhibited by KCN (1 mM), thus excluding a significant contribution by alternative oxidase pathway [52].

**Figure 2:**
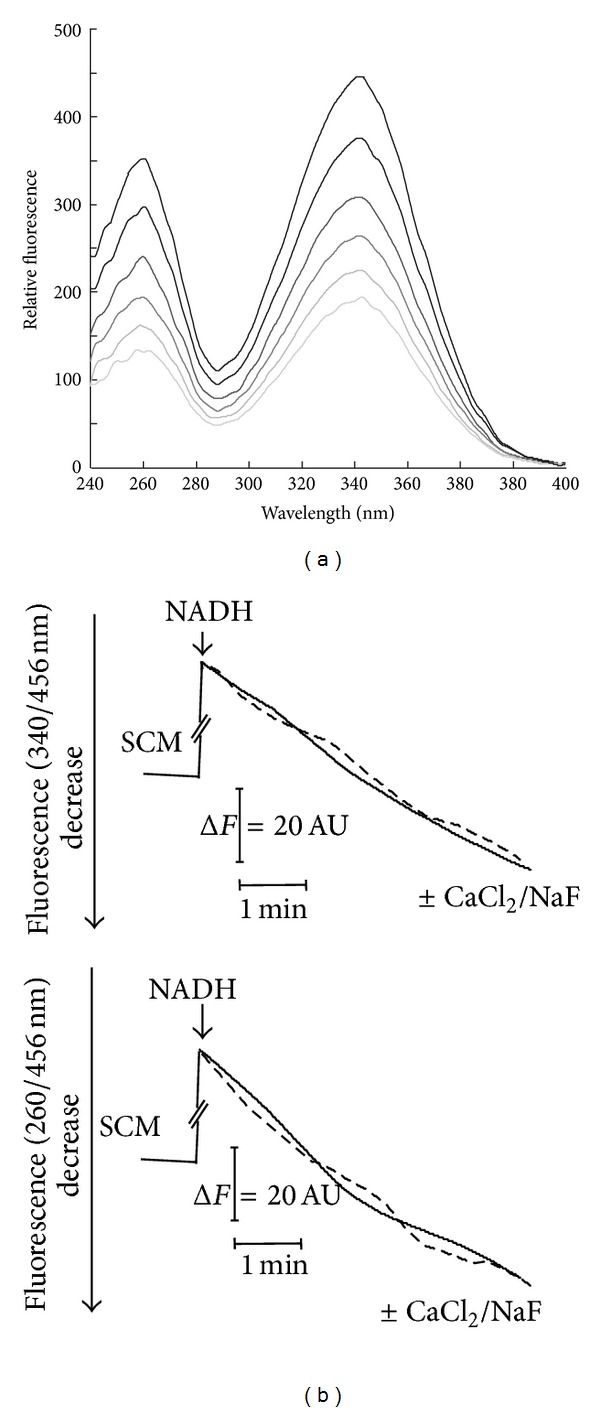
Fluorimetric evidence of NADH cleavage catalyzed by solubilized SCM. (a) SCM (0.025 mg protein) were added to NADH (5 *μ*M) in the standard medium supplemented with KCN (1 mM) and the reaction followed at 25°C, essentially as described in Section 2. NADH fluorescence excitation spectra (emission wavelength set at 456 nm) were recorded at different incubation times. (b) NADH fluorescence decrease, induced by freezing-thawing rupture of SCM, was continuously measured at both the wavelength pairs 260/456 and 340/456 nm, under the experimental condition described in (a). Where indicated NaF or CaCl_2_ (1 mM each) were added (dotted lines).

To better investigate the fate of externally added NADH, SCM were ruptured by freezing-thawing cycles and added to NADH (5 *μ*M) in the absence and in the presence of KCN (1 mM) (Figure 2(a)). Fluorescence excitation spectra of the suspension were registered for 10 min, with the emission wavelength set at 456 nm. As expected, two major emission fluorescence peaks are found (at 260 and 340 nm, resp.) due to the reduced form of the coenzyme, with the corresponding fluorescence intensities decreasing concomitantly in the course of NADH oxidation at both 260 nm and 340 nm. From the values of fluorescence decrease, a NADH oxidation rate of 520 nmol · min^−1^ · mg^−1^ protein was calculated as reported in Section 2 (data not shown). KCN (1 mM) addition to ruptured SCM reduced the rate of NADH disappearance to less than 8% of that measured in its absence (Figure 2(a)). Under this experimental conditions, the rate of fluorescence decrease at wavelength pairs 340/456 nm (essentially due to the KCN-insensitive NADH oxidation rate) became lower than that measured at 260/456 nm. 

A possible explanation for this asymmetry derived from the hypothesis that oxidation is not the sole process responsible for NADH disappearance in the suspension, with a possible contribution due to putative hydrolytic processes. In this case a NADH hydrolysis rate equal to 14 nmol NADH hydrolyzed · min^−1^ · mg^−1^ protein was calculated by applying (4) reported in Section 2, while NADH oxidation rate was reduced at 48 nmol · min^−1^ · mg^−1^ protein. In three independent experiments using different mitochondrial preparations, a mean rate of 14.6 ± 1.1 nmol NADH hydrolyzed · min^−1^ · mg^−1^ protein was calculated (with NADH oxidation rate of 34 ± 12 nmol · min^−1^ · mg^−1^ protein). Similar results were obtained when the fluorescence decrease was continuously measured at both the wavelength pairs 260/456 nm and 340/456 nm, respectively. Typical traces are reported in Figure 2(b) (straight lines).

The specific NADH hydrolytic activity measured in the mitochondrial fraction resulted almost equal to that found in whole sphero (14.1 ± 1.6 nmol · min^−1^ · mg^−1^ protein); this allows us to propose a mitochondrial localization for NADH destroying enzyme, even if not exclusive. The already characterized hydrolase which is known to cleave NADH, namely, Npy1p (see Section 1), seemed to be peroxisomal [28]. With the aim of identifing the NADH destroying enzyme, we queried a number of protein sorting signal prediction programs (i.e., iPSORT, TargetP, and MITOPROT), thus finding a possible mitochondrial localization for this protein.

To understand whether the mitochondrial NADH hydrolytic activity was due to the NUDIX hydrolase Npy1p, the effects of both NaF (1 mM) and CaCl_2_ (1 mM), powerful inhibitors of the peroxisomal enzyme, were tested (Figure 2(b)). Since they clearly did not inhibit the NADH hydrolysis rate (dotted lines), we exclude that the activity we revealed was due to a putative mitochondrial isoform of this enzyme. Independently from an auspicial protein identification, these experiments represent the first evidence in favor of the existence of a novel mitochondrial NAD(H) destroying activity. Nevertheless, the rapid fluorimetric method used prevents a detailed kinetic characterization.

**Figure 3:**
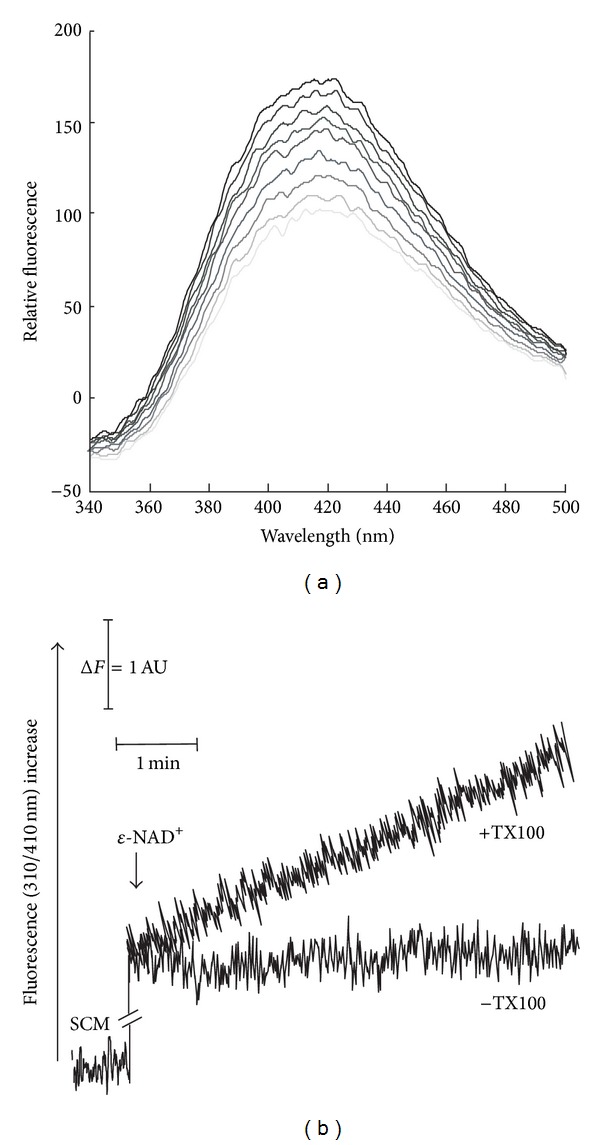
Fluorimetric evidence of *ε*-NAD^+^ cleavage catalyzed by solubilized SCM. (a) SCM (0.3 mg protein), solubilized with TX100, were added to *ε*-NAD^+^ (50 *μ*M) in the standard medium and the reaction followed at 25°C, essentially as described in Section 2. *ε*-NAD^+^ emission spectra (at excitation wavelength 310) were monitored at different incubation times. (b) *ε*-NAD^+^ fluorescence decrease was continuously monitored at 310/410 nm under the same experimental condition described in (a) using intact (−TX100) or solubilized SCM (+TX100).

A simpler investigation of NAD-destroying activities makes use of a NAD^+^ fluorescent analog, namely, nicotinamide 1,N^6^-ethenoadenine dinucleotide (*ε*-NAD^+^) [50, 51]. This assay was specifically useful to investigate whether purified SCM can hydrolyze also the oxidized form of the pyridine coenzyme. In Figure 3(a) a typical *ε*-NAD^+^ (50 *μ*M) emission spectrum is reported, as revealed at pH 7.5, in the 340–500 nm range, with excitation wavelength set a 310 nm. As expected, it reveals a characteristic peak at 410 nm, whose intensity linearly depends on *ε*-NAD^+^ concentration. Addition of SCM (previously solubilized with TX100) induced an increase in fluorescence emission at 410 nm, as a linear function of time up to 20 min. Since the *ε*-adenine ring specific fluorescence constant is about ten-fold higher than that of the *ε*-NAD^+^, the observed fluorescence increase strongly suggests that *ε*-NAD^+^ was cleaved with a rate equal to 0.23 nmol *ε*-NAD^+^ cleaved · min^−1^ · mg^−1^ protein. In Figure 3(b) the fluorescence changes of *ε*-NAD^+^ (50 *μ*M) suspensions, following the addition of solubilized SCM, were continuously measured, at the fixed excitation and emission wavelengths (set at 310 nm and 410 nm, resp.), corresponding to an initial rate of hydrolysis equal to 0.25 nmol · min^−1^ · mg^−1^ protein (+TX100). No increase was observed when freshly isolated intact SCM were used (−TX100), thus suggesting an intramitochondrial localization for NAD^+^-cleaving activity.

**Figure 4:**
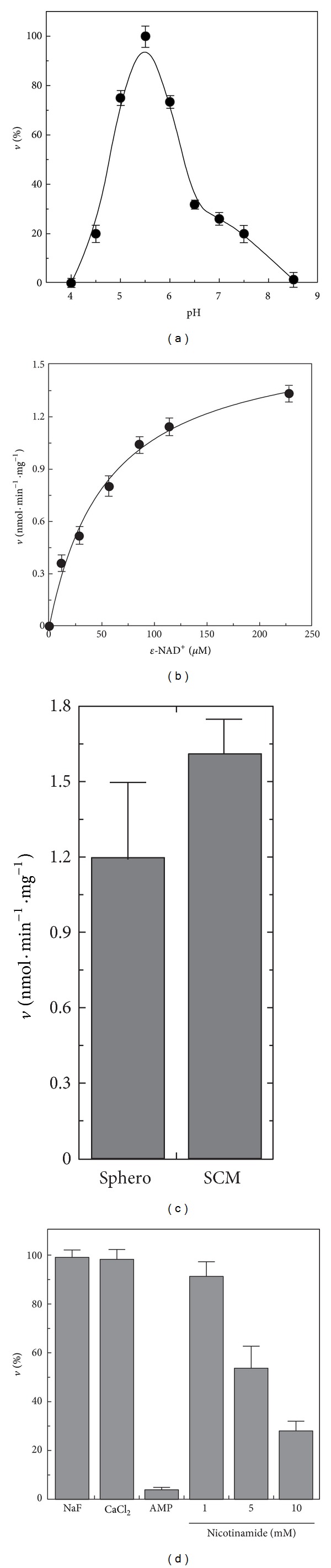
Some features of *ε*-NAD^+^ cleavage. (a) The pH profile of the rate of *ε*-NAD^+^ (100 *μ*M) cleavage, catalyzed by solubilized SCM (0.3 mg protein), was fluorimetrically measured as described in Section 2. Use was made of 100 mM acetate/acetic acid and 50 mM Tris/HCl buffering mixtures (supplemented with 5 mM MgCl_2_), whose pH was adjusted to the desired value. A specific calibration curve was obtained at each pH value as described in Section 2. The values are reported as percentage of the maximum rate (i.e., 1.6 nmol · min^−1^ · mg^−1^), arbitrarily set equal to 100%. (b) The dependence of the rate of *ε*-NAD^+^ cleavage on substrate concentration was measured using solubilized SCM (0.3 mg protein) at pH 6. (c) The rate of *ε*-NAD^+^ (200 *μ*M) cleavage catalyzed by TX100-solubilized sphero and SCM (0.3 mg protein each) was measured at pH 6. (d) The sensitivity of *ε*-NAD^+^ (50 *μ*M) cleavage (catalyzed by solubilized SCM, 0.3 mg protein) towards NaF, CaCl_2_, AMP (1 mM each), and nicotinamide (at the indicated concentrations) was measured at pH 6.

The dependence of the rate of cleavage of *ε*-NAD^+^ catalyzed by solubilized SCM was determined in the pH range 4.5–10, using 100 mM acetate/acetic acid and 50 mM Tris/HCl buffering mixtures (Figure 4(a)). *ε*-NAD^+^cleavage was found to occur with a bell-shaped profile with the maximum rate measured at pH 5.5.

To gain some insights into the substrate affinity, the dependence of the rate of *ε*-NAD^+^ was studied at pH 6 as a function of the substrate concentration (Figure 4(b)). Hyperbolic characteristic was found, with saturation kinetics analyzed by the Michaelis-Menten equation, using the *GraFit* software (see Section 2). Thus, *K*_*m*_ and *V*_max⁡_ values equal to 56 ± 8.9 *μ*M and 1.7 ± 0.1 nmol · min^−1^ · mg^−1^ of mitochondrial protein, respectively, were calculated from the data in Figure 4(b). In three independent experiments using different mitochondrial preparations, a mean rate of 1.6 ± 0.2 nmol *ε*-NAD^+^ hydrolyzed · min^−1^ · mg^−1^ protein was found, using *ε*-NAD^+^ (200 *μ*M).

The specific *ε*-NAD^+^ hydrolytic activity was also measured in solubilized sphero, under the same experimental conditions. As reported in Figure 4(c), the specific *ε*-NAD^+^ hydrolytic activity in sphero is similar to that measured in SCM. This finding, as previously observed for NADH hydrolytic activity, suggests a preferential, but not exclusive, mitochondrial localization for this enzyme.

As previously observed for NADH hydrolyzing activity, the mitochondrial *ε*-NAD^+^ hydrolytic activity was not inhibited by both NaF (1 mM) and CaCl_2_ (1 mM) (Figure 4(d)), thus again excluding that a NUDIX hydrolase was responsible for this activity. Conversely, the mitochondrial NAD^+^-cleavage activity was totally inhibited by AMP (1 mM) but also significantly inhibited by nicotinamide, with an IC_50_ of about 5 mM (Figure 4(d)).

This last experiment strongly supports the proposal that a nicotinamide-sensitive pyridine nucleotide-destroying enzyme is enriched in SCM, even if, unfortunately, it did not allow to precisely identify the product of mitochondrial NAD(H) hydrolysis. Nevertheless, the strong inhibition by AMP allows us to postulate that the dinucleotide was broken at the level of pyrophosphate bond, as already demonstrated for certain animal and plants FAD pyrophosphatases (FADppase, EC 3.6.1.18) [54, 55]. 

Particular attention merits the finding that NAD^+^ degradation, as well as FAD degradation (see below), strictly depends on pH, which in yeast is highly dynamic [56]; thus, we expect that local changes in intramitochondrial pH may have profound influence on the availability of intramitochondrial pyridine and flavin cofactors. Under respiratory conditions, an intramitochondrial pH value ranging from 7.7 to 7.3 during the different growth phases was experimentally evaluated [57], presumably accompanied by a very low cofactor degradation, as shown by measurements performed here. Thus, under physiological conditions, respiratory-chain-driven proton pumping prevents cofactor hydrolysis by generating a transmembrane pH gradient, negative inside.

Mild uncoupling [58] or various stress conditions [57] induce a severe decrease in intramitochondrial pH value, which plays a central role, together with vacuoles, in controlling cytosolic pH [56, 59, 60]. Under these conditions we expected an increase in the rate of NAD^+^, as well as FAD degradation (see below), which in turn may regulate redox homeostasis, cell growth, and defense responses [61, 62].

Since the contribution of mitochondrial NAD(H) degrading enzyme activity on the economy of NAD(H) homeostasis and, in turn, on cell longevity and stress resistance is still unexplored, our future goal will be to identify acidic mitochondrial NAD pyrophosphatase in yeast.

**Figure 5:**
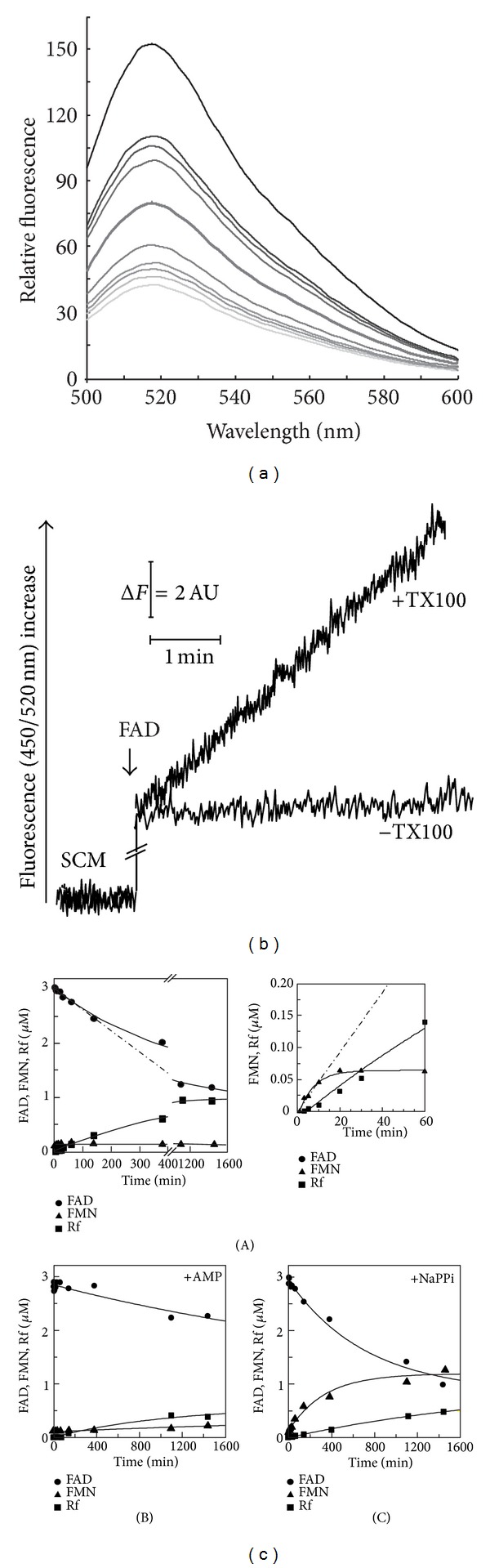
FAD pyrophosphatase in solubilized SCM. (a) SCM (0.05 mg protein), solubilized with TX100, were incubated under the experimental conditions described in Section 2. The reaction was started by FAD (3 *μ*M) addition to the mitochondrial suspension and followed at 25°C. FAD emission spectra (at excitation wavelength 450 nm) were monitored at different incubation times. (b) FAD fluorescence decrease was continuously monitored at 450/520 nm under the same experimental condition described in (a) using intact (−TX100) or solubilized SCM (+TX100). (c) Extracts of the FAD hydrolysis reaction mixture were taken at different incubation times and analyzed via HPLC, as described in Section 2 with measurements of FAD, FMN, and Rf concentrations. The value obtained were plotted against the incubation time after FAD addition. FAD hydrolysis reaction was carried in the absence (panel (A)) or in the presence of AMP (1 mM) (panel (B)) or NaPPi (1 mM) (panel (C)). The inset in (panel (A)) is an enlargement of the first 60 min of the plot reported in the same panel.

As far as FAD degradation is concerned, the occurrence of AMP-inhibited FAD hydrolysis in isolated intact mitochondria was for the first time demonstrated in rat liver [38] and, more recently, in *S. cerevisiae* [40]. In order to understand whether FADppase, described in [40], could also be responsible for the mitochondrial NAD(H) hydrolyzing activity observed in our mitochondrial preparation, FADppase was characterized in some detail. Thus, FAD (3 *μ*M) was added to solubilized SCM and flavin fluorescence emission spectra (with excitation wavelength set at 450 nm) registered at pH 7.5, as a function of time. As expected, an increase in the 500–600 nm wavelength range with a peak at 520 nm was observed in Figure 5(a), due to the conversion of FAD into FMN/Rf, with a rate of fluorescence increase, measured as described in Section 2, corresponding to 0.28 nmol FAD hydrolyzed · min^−1^ · mg^−1^ protein.

In Figure 5(b), FAD (3 *μ*M) fluorescence was continuously measured at the fixed excitation and emission wavelengths (set at 450 nm and 520 nm, resp.). No fluorescence increase was observed after addition of intact and coupled SCM (−TX100). When FAD was added to solubilized SCM an increase was observed with a rate equal to 0.30 nmol FAD hydrolyzed · min^−1^ · mg^−1^ protein (+TX100). In seven experiments performed with different mitochondrial preparations, a mean rate of 0.32 ± 0.05 nmol FAD hydrolyzed · min^−1^ · mg^−1^ protein was measured. FAD hydrolysis could be revealed only when SCM were solubilized by TX100 and in this aspect SCM are different from rat liver mitochondria.

In order to characterize the products of FAD hydrolysis catalyzed by solubilized mitochondria, under the same experimental conditions described in Figure 5(a), extracts of the suspension were taken at different incubation times and analyzed via HPLC, as described in Section 2, with measurements made of FAD, FMN, and Rf amount in the suspension.

Solubilized SCM added with FAD (3 *μ*M) were incubated either in the absence (panel (A)) or in the presence of AMP (panel (B)). In a parallel assay the effect of NaPPi (panel (C)), which was proved to prevent FMN hydrolysis in SCM [31], was also tested.

As shown in Figure 5(c) (panel (A)), FAD amount was found to decrease with a constant initial rate of 0.29 nmol FAD hydrolyzed · min^−1^ · mg^−1^ protein, in agreement with the fluorimetric measurements, and then it reached a plateau. Concomitantly with FAD disappearance, a significant increase in FMN amount was initially observed with a constant rate of 0.30 nmol · min^−1^ · mg^−1^ protein in the first 10 min, in a fairly good agreement with a stoichiometric ratio of 1 : 1 with the FAD amount decrease (see inset). At longer incubation time, Rf, that was not initially detectable, appeared in the suspension (see inset) and it increased linearly up to 60 min (with a rate of 0.10 · min^−1^ · mg^−1^ protein), thus prevailing over FMN (panel (A)) which remained relatively constant (at about 0.06 *μ*M). Since FAD hydrolysis rate does not depend on the rate of FMN into Rf conversion in the first 10 min (that is, the step catalyzed by FADppase is the limiting step of mitochondrial FAD degradation pathway), kinetics measurements of FADppase reaction were performed consistently by fluorimetric method.

As expected, the rate of FAD hydrolysis was totally inhibited by AMP (1 mM); neither FMN nor Rf was found to appear during the incubation time (panel (B)). Conversely, in the presence of NaPPi (1 mM), the rate of FAD hydrolysis resulted unchanged, with a notable increase in FMN (with an initial rate of 0.30 nmol · min^−1^ · mg^−1^ proteins), which was slowly accompanied by Rf appearance with an 85% reduced rate, in comparison to the rate measured in the absence of NaPPi (panel (C)). 

When the mitochondrial FAD (3 *μ*M) hydrolysis was studied at pH 6 (i.e., the optimum of *ε*-NAD^+^ hydrolytic activity), FAD disappearance was found to occur with a 2.5-fold higher rate and to be still in a good agreement with a stoichiometric ratio of 1 : 1 with the appearance of FMN, that is the preeminent species at this pH value, whereas the rate of Rf appearance is extremely low (0.02 nmol · min^−1^ · mg^−1^ mitochondrial proteins).

**Figure 6:**
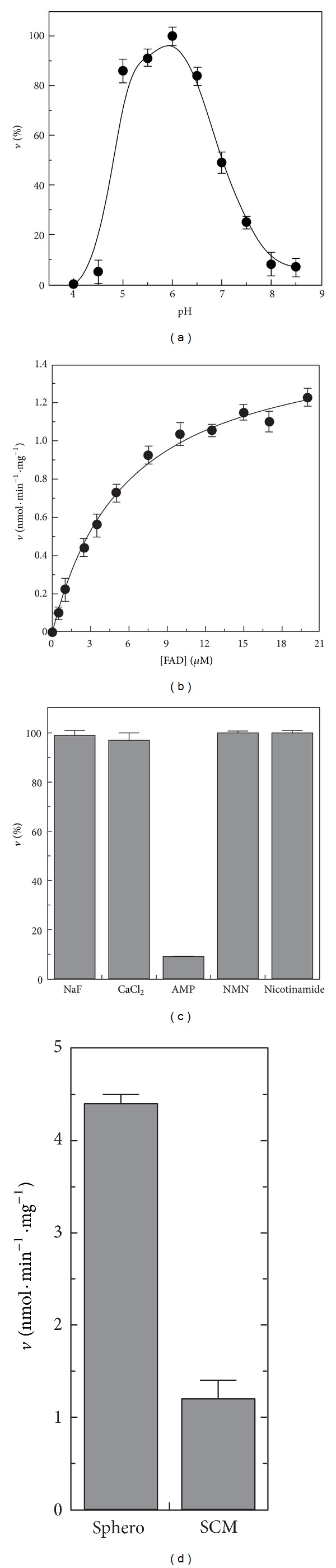
Some features of FAD pyrophosphatase. (a) The pH profile of the rate of FADppase, catalyzed by solubilized SCM (0.1 mg protein) in the presence of FAD (10 *μ*M), was fluorimetrically measured as described in Section 2. Use was made of 100 mM acetate/acetic acid and 50 mM Tris/HCl buffering mixtures (with 5 mM MgCl_2_), whose pH was adjusted to the desired value. A specific calibration curve was obtained at each pH value in Section 2. The values are reported as percentage of the maximum rate (i.e., 1.05 nmol · min^−1^ · mg^−1^ protein), arbitrarily set equal to 100%. (b) The dependence of the rate of FADppase on substrate concentration was measured at pH 6 using solubilized SCM (0.1 mg protein). (c) The sensitivity of FADppase, catalyzed by solubilized SCM (0.1 mg) in the presence of FAD (10 *μ*M), towards NaF, CaCl_2_, AMP, NMN (1 mM each), and nicotinamide (5 mM) was measured at pH 6. (d) The rate of FAD (10 *μ*M) cleavage, catalyzed by solubilized sphero and SCM (0.1 mg protein each), was measured at pH 6.

Consistently, the rate of FAD cleavage catalyzed by solubilized SCM, fluorimetrically determined in the pH range 4.0–10 in Figure 6(a), was found to occur with a bell-shaped profile with the maximum rate measured at pH 6 (being equal to 1.05 nmol · min^−1^ · mg^−1^ protein, when FAD 10 *μ*M was used as substrate). This pH profile is quite different from that described in [40], whose optimum activity was at alkaline pH. Thus, the dependence of FAD hydrolysis rate on substrate concentration was studied at pH 6 (Figure 6(b)). Hyperbolic characteristic was found with saturation kinetic, analyzed by the Michaelis-Menten equation, using the *GraFit* software (see Section 2). Thus, *K*_*m*_ and *V*_max⁡_ values equal to 5.9 ± 0.5 *μ*M and 1.6 ± 0.1 nmol · min^−1^ · mg^−1^ of mitochondrial protein, respectively, were calculated from the data in Figure 6(b). A *K*_*m*_ value equal to 10 ± 2 *μ*M was determined at pH 7.5 (data not shown).

These results confirm that in SCM, a FADppase exists and it is able to catalyze the cleavage of the diphosphate bound in dinucleotides. This enzyme is inhibited by AMP and stimulated by acidic pH value. A putative mitochondrial FMN phosphohydrolase activity is involved in FMN dephosphorylation; it is specifically inhibited by NaPPi and its activity is significantly reduced at acidic pH value.

As regards inhibition of FAD hydrolysis, the inorganic compounds NaF (1 mM) and CaCl_2_ (1 mM), differently from AMP (1 mM), did not reduce FAD hydrolytic activity in SCM (Figure 6(c)), as already observed for *ε*-NAD^+^ hydrolysis, thus excluding that FMN is produced by a putative mitochondrial NUDIX hydrolase. 

Quite interestingly, differently from *ε*-NAD^+^ hydrolysis, nicotinamide (as well as NMN) did not affect FAD hydrolysis rate. Therefore, we may conclude that mitochondrial NAD(H) and FAD destroying activities are different. Another difference between mitochondrial NAD(H) and FAD destroying activities resides in the observation that specific FAD hydrolytic activity catalyzed by solubilized SCM corresponded to about only 27% of that measured in the sphero (Figure 6(d)). Taking into account the extramitochondrial contaminations present in our mitochondrial preparations (see Figure 1(a)), it represents about 10% of the total activity detectable in the sphero. A similar distribution ratio was also found for other mitochondrial enzymes, that is, RFK and FADS [29, 31]. Presumably, other quite active FADppases are localized in the extramitochondrial compartments.

**Figure 7:**
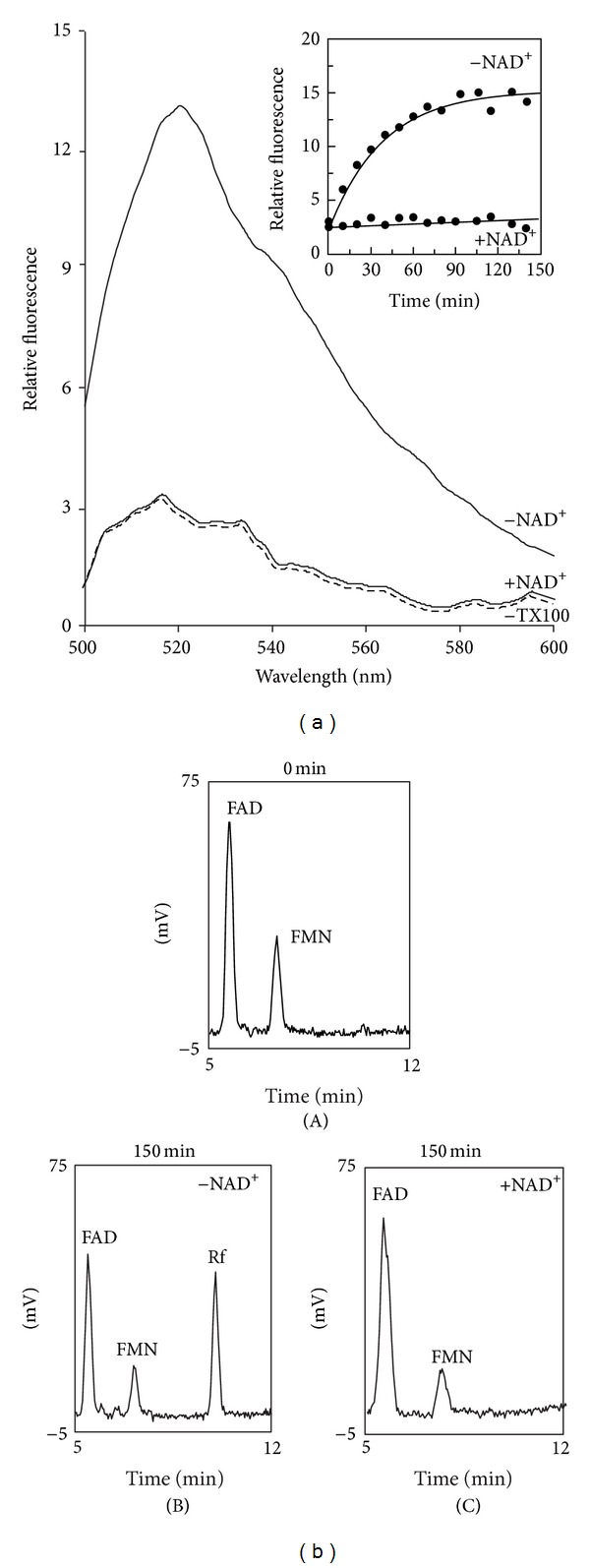
Endogenous FAD hydrolysis by isolated SCM induced by TX100; inhibition by NAD^+^. (a) SCM (0.1 mg protein) were incubated under the experimental conditions described in Section 2. Fluorescence emission spectra (excitation wavelength at 450 nm) of the suspension were recorded after 150 min in the absence (−TX100, dotted line) or in the presence of TX100 (0.1%). Where indicated, NAD^+^ (1 mM) was also added. In the inset, the fluorescence intensity, measured at 520 nm, as obtained after TX100 addition, either in the absence or in the presence of NAD^+^, was plotted against the incubation time. (b) FAD, FMN, and Rf in TX100 treated mitochondrial suspension extracts were revealed by means of HPLC measurements, performed as described in Section 2, following 0 (panel (A)) or 150 min incubation time in the absence (panel (B)) or presence (panel (C)) of NAD^+^ (1 mM).

In a first attempt to determine the physiological role for the intramitochondrial FADppase, the possibility that this enzyme can play a major role in the intramitochondrial FAD degradation was studied by both fluorescence and HPLC experiments. A typical fluorimetric experiment is reported in Figure 7(a). At initial time, intact mitochondria showed a typical endogenous flavin emission spectrum with a peak at 520 nm (excitation wavelength set at 450 nm) (−TX100, dotted line). No increase in flavin fluorescence was observed when coupled and intact SCM were used. When SCM were solubilized with TX100 an increase in the 500–600 nm wavelength range was observed (−NAD^+^). This strongly suggests that endogenous FAD has been hydrolyzed to FMN/Rf. Endogenous flavin fluorescence was found to increase as a linear function of time up to 30 min with a rate equal to 7 pmol endogenous FAD hydrolyzed · min^−1^ · mg^−1^ protein (see inset). It should be noted that under our experimental conditions less that 20% of total mitochondrial FAD amount was hydrolyzed, presumably due to an inhibition by FAD hydrolysis product(s) or because the remaining FAD was bound to holoflavoprotein and thus protected by hydrolytic activity.

Since FADppase is located inside mitochondria the stimulatory effect of the detergent could not be easy explained, unless some internal metabolite, whose concentration is high in the matrix of intact organelles and diluted following membrane rupture, is able to inhibit FADppase. In this hypothesis we tested a possible inhibitory effect by NAD^+^ (1 mM), which was found to completely inhibit endogenous FAD to FMN conversion (+NAD^+^). 

In the same experiment, the hydrolysis of endogenous FAD was confirmed by HPLC measurements of FAD, FMN, and Rf amounts before and after TX100 addition (Figure 7(b)). In the absence of TX100 the mitochondrial amounts of FAD and FMN were about 172 and 26 pmol · mg^−1^ protein, respectively, and no Rf was detected (panel (A)). As a result of TX100 addition, FAD and FMN were found to decrease, with significant appearance of Rf (panel (B)). Rf content was found to increase up to 20 min in a fairly good stoichiometric ratio (1 : 1) with FAD decrease (data not shown). The rate of FAD hydrolysis (i.e., Rf appearance) was equal to 7.2 pmol · min^−1^ · mg^−1^ protein, in good agreement with fluorescence measurements. Rf appearance was strongly prevented by NAD^+^ (panel (C)).

**Figure 8:**
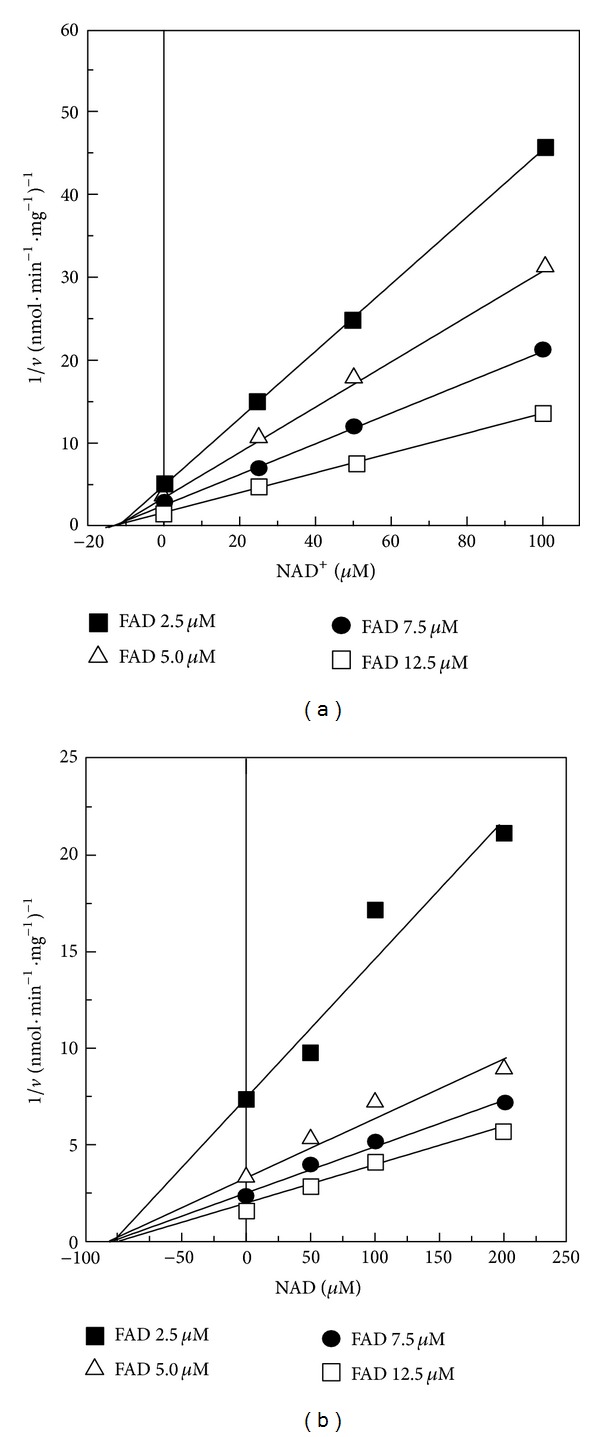
NAD^+^ and NADH inhibition of FAD pyrophosphatase. The rate of FAD hydrolysis (*v*) was fluorimetrically measured in the standard medium at pH 7.5 with FAD at the indicated concentrations (2.5, 5, 7.5, or 12.5 *μ*M) and solubilized SCM (0.05 mg protein). NAD^+^ or NADH was added in the FAD degradation reaction mixture at the concentration indicated. When NADH sensitivity was tested, KCN (1 mM) was added to the reaction mixture. The Dixon plots of the inhibition by NAD^+^ and NADH are reported in panels (a) and (b), respectively.

The effect of NAD^+^ was confirmed on exogenous FAD hydrolysis by HPLC (data not shown) and the nature of the inhibition studied fluorimetrically (Figure 8(a)). FAD hydrolysis sensitivity to NAD^+^ was consistent with a noncompetitive inhibition, as shown in the Dixon plot with a *Ki* value equal to 10 *μ*M, calculated from the data in Figure 8(a).

Adenosine and derived compounds (i.e., ADP-ribose, Ap_5_A, and CoA) and guanosine nucleotides, but not nicotinamide and NMN, are able to reduce FAD hydrolysis rate (data not shown); therefore we feel that the purine moiety is relevant for inhibitor recognition by FADppase. Consistently, NADH is also able to inhibit in a noncompetitive way the rate of FAD hydrolysis, but, interestingly, the *Ki* differed for an order magnitude, being equal to 100 *μ*M for NADH, as calculated from the data reported in Figure 8(b).

**Figure 9:**
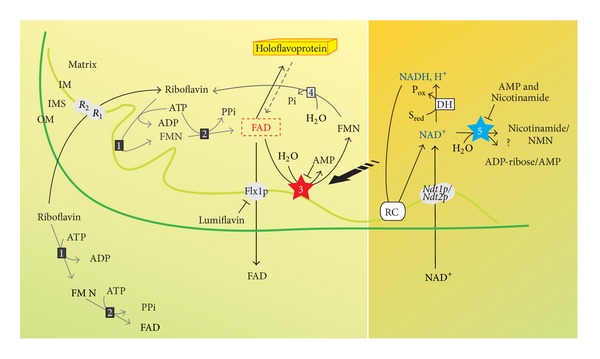
FAD and NAD homeostasis in SCM. The scheme summarized the functional studies described in this and other previous papers [23, 31, 32, 53]. R_1_/R_2_, mitochondrial Rf transporter; Flx1p, mitochondrial FAD exporter; *Ndt1p/Ndt2p*, mitochondrial NAD^+^ transporter; (1), riboflavin kinase (EC 2.7.1.26); (2), FAD synthase (EC 2.7.7.2); (3), FAD pyrophosphatase (EC 3.6.1.18); (4), FMN phosphohydrolase (EC 3.1.3.2); (5), NAD(H) cleaving enzyme; DH, NAD(H)-dependent dehydrogenase; RC, respiratory chain.

These results demonstrate that FAD hydrolyzing enzyme could discriminate between the redox status of pyridine nucleotides, as depicted in Figure 9. It is well known that a high electron pressure, namely, an increase in NADH/NAD^+^ ratio, is a condition favoring ROS generation which can be contrasted by an efficient respiratory chain (which is flavin dependent) and by defense systems (some of which are flavoenzymes) [6]. According to the measurements performed here, reduction of NAD^+^ to NADH is expected to favor hydrolysis of free FAD, since the oxidized form of the pyridine cofactor is more powerful as inhibitor (lower *Ki*) than the reduced form (higher *Ki*), at least in ruptured SCM. Thus, we can speculate that reducing conditions are accompanied by a more rapid local “recycling” of FAD, presumably deriving from holoflavoprotein turnover. This situation could allow a more rapid intramitochondrial assembly of novel flavoproteins (mainly components of the respiratory chain, like SDH [32, 63]) and saving of cytosolic Rf level which is necessary for enzymatic ROS defense. 

Whether or not flavin-coenzymes homeostasis is modulated *in vivo* by pyridine nucleotide redox status inside SCM as postulated and summarized in the scheme in Figure 9, is an interesting interrogative that we would like to address in the next future.

## 4. Conclusions

Here we proved the ability of SCM to catalyze NAD(H) and FAD hydrolysis, via enzymatic activities which are different from the already characterized NUDIX hydrolases. 

The differential inhibition by the oxidized and reduced form of NAD toward the mitochondrial FADppase, together with the ability of mitochondrial FADppase to metabolize endogenous FAD, presumably deriving from mitochondrial holoflavoproteins destined to degradation, allows for proposing a novel possible role of mitochondrial NAD redox status in regulating FAD homeostasis and, possibly, flavoprotein degradation in *S. cerevisiae*.

## Abbreviations

FAD: Flavin adenine dinucleotide
NAD: Nicotinamide adenine dinucleotide
Rf: Riboflavin
RFK: Riboflavin kinase
FADS: FAD synthase
SCM: *Saccharomyces cerevisiae* mitochondria
sphero: Spheroplasts
PDC: Pyruvate decarboxylase
AP: Alkaline phosphatase
D-AAOX: D-amino acid oxidase
CS: Cytrate synthase
FUM: Fumarase
TX100: Triton X-100
RCI: Respiratory control index
FADppase: FAD pyrophosphatase.
